# Algorithms and Methods for the Fault-Tolerant Design of an Automated Guided Vehicle

**DOI:** 10.3390/s22124648

**Published:** 2022-06-20

**Authors:** Ralf Stetter

**Affiliations:** Department of Mechanical Engineering, Ravensburg-Weingarten University (RWU), 88250 Weingarten, Germany; ralf.stetter@rwu.de

**Keywords:** fault-tolerant control, fault-tolerant design, automated guided vehicle

## Abstract

Researchers around the globe have contributed for many years to the research field of fault-tolerant control; the importance of this field is ever increasing as a consequence of the rising complexity of technical systems, the enlarging importance of electronics and software as well as the widening share of interconnected and cloud solutions. This field was supplemented in recent years by fault-tolerant design. Two main goals of fault-tolerant design can be distinguished. The first main goal is the improvement of the controllability and diagnosability of technical systems through intelligent design. The second goal is the enhancement of the fault-tolerance of technical systems by means of inherently fault-tolerant design characteristics. Inherently fault-tolerant design characteristics are, for instance, redundancy or over-actuation. This paper describes algorithms, methods and tools of fault-tolerant design and an application of the concept to an automated guided vehicle (AGV). This application took place on different levels ranging from conscious requirements management to redundant elements, which were consciously chosen, on the most concrete level of a technical system, i.e., the product geometry. The main scientific contribution of the paper is a methodical framework for fault-tolerant design, as well as certain algorithms and methods within this framework. The underlying motivation is to support engineers in design and control trough product development process transparency and appropriate algorithms and methods.

## 1. Introduction

Throughout the industry, a general trend towards more complex and interconnected technical systems can be observed. Simultaneously, the reliability requirements are more difficult to satisfy than ever. Increased functional options and an enhanced system performance are required in a global competition. This may lead to enhanced customer satisfaction, but it also leads to an increasing number of possibilities for faults. It is important to note that a fault can be understood as a deviation of one or more characteristic parameters or properties of the respective system from the acceptable, regular condition, which is not permitted [1]; this distinguishes a fault from a failure, which, in general, indicates a catastrophic event. Under the notion “fault-tolerant control” (FTC), algorithms and systems are summarized, which are intended to accommodate possible effects of appearing faults and to prevent failure. Fault-tolerant control can be facilitated and simplified through certain design characteristics, for instance an easy access to energy flows that need to be measured for the detection of faults. Moreover, some design aspects may enlarge the fault-tolerance independently from fault-tolerant control, for instance, redundant elements or over-actuation. The term “fault-tolerant design” (FTD) can be used for summarizing both types of approach; the concept, models and methods of FTD is the scope of the later sections of this paper.

The main objective of this paper is the explanation and discussion of a methodical framework for fault-tolerant design as well as algorithms and methods within this framework. One of the main goals of fault-tolerant design is the improvement of the controllability of technical systems; the state of the art of this important research field concerning the control of AGVs is discussed in the next subsection.

### 1.1. State of the Art in Fault-Tolerant Control

Several researchers around the globe intend to enlarge the theoretical knowledge in the area of fault-tolerant control, to synthesize innovative algorithms and systems as well as to apply them to real-life problems. A synoptic review of the results is given in the publications of [1,2,3,4]. Current investigations expand the knowledge in the field of FTC. One important field is fault detection, research is concerned with the extraction of nonlinear features using a sparse auto-encoder and a sparse restricted Boltzmann machine [5] and a multi-resolution analysis of traveling waves in direct current (DC) micro-grids [6]. One main field is fault estimation; researchers investigate sliding mode observers [7], proportional-integral observers [8] and the possibility of relaxed assumptions [9]. Another important topic is state estimation; research activities employ for instance interacting multiple model estimation algorithms [10]. Rule-based expert systems again receive attention such as in the fault diagnosis of mechanical systems [11]. Further research is involved with diagnostic row reasoning methods [12]. The integration of the techniques of artificial intelligence such as neural networks is investigated using the examples of spacecrafts [13], distributed power generators [14] and transmission lines [15], industrial robots [16] and bearings [17]. Additionally, investigations of fault-tolerant control systems in certain application fields are currently expanded such as in the field of underwater vehicles [18], octorotor UAVs [19], regional aircrafts [20], chemical reactors [21], wind turbines [22], fault-tolerant permanent magnet motors [23] and the power steering of forklifts [24]. Furthermore, reviews in certain fields are published that supplement the reviews mentioned above; these reviews concern the fault diagnosis of machines with small and imbalanced data [25], fault prediction and location methods in electrical energy distribution networks [26], rotating machinery fault detection and diagnosis applying deep domain adaptation [27] as well as intelligent fault-diagnosis for high-speed trains [28].

A frequent cause for faults can be network issues, such as packet losses. Consequently, the communication aspects of controlling AGVs also play an important role and require in-depth scientific work. A large number of autonomous entities (e.g., AGVs) may be operating in one production site and the underlying networks frequently consist of a variety of wide-area and local-area networks (5G, WiFi, field buses, etc.) [29]. Several research groups are working on this topic. Important research works concern the evaluation of architectural patterns for the remote control of AGVs [29], live visualizations for remote AGV operation [30] and formation control of AGVs in the presence of package losses [31]. As reflected in the research of Sedghi et al. [31], the influence of faults can be especially high, when AGVs need to interact in order to achieve a common task. A very important area for scientific investigation covers the formation control of AGVs. This kind of control for collaborative transportation, commonly also referred to as collective transport, is a key challenge for cooperative AGVs [32]. Current research investigates communications-based formation control [32] and consensus based control for dynamic environments [33]. A promising future research field will be fault-tolerant formation control—research concerning formation control in the presence of package losses points in this direction [31]. In formation control many uncertain conditions are present and formation control problem with external interference should be studied in the future [34].

The analysis of research concerning fault-tolerant control makes it obvious that powerful approaches are being developed, but that all rely on reliable sensors and actuators and an appropriate design of the technical system. This issue is discussed in detail in this paper based on an application example.

### 1.2. Application Scenario

**Figure 1:**
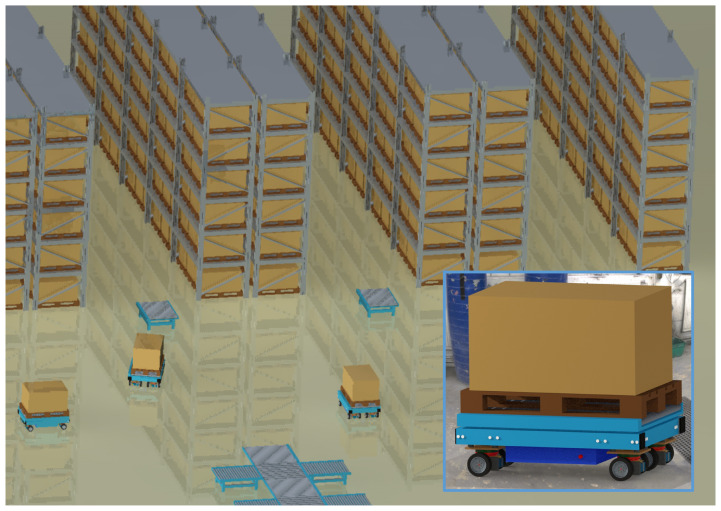
Automated guided vehicle (AGV) and operation scenario.

The main part of this paper discusses fault-tolerant design using the example of an AGV. The AGV under consideration realizes a unique torque steering concept by employing four mechanically independent drive modules. Figure 1 shows this kind of AGV and a typical operation scenario in a warehouse.

### 1.3. Structure of the Paper

The central goal of this paper is the presentation of a methodical framework for fault-tolerant design. In order to form a basis for the explanation and discussion, Section 2 explains the methodical background of this framework. The central section of this paper is Section 3, which discusses design aspects and considerations that facilitate a fault-tolerant design of the AGV. The paper closes with a conclusion and summary.

## 2. Fault-Tolerant Design

This section explores the methodical background of a framework for fault-tolerant design. Until today, several approaches to enable the accommodation of faults directly and indirectly through design characteristics were reported. However, the integration in product development processes was usually not a focus. Therefore, this section firstly summarizes important research works and integrates fault-tolerant design in model-based systems engineering (MBSE), which is the most common approach in industrial companies.

Fault-tolerant design is a notion that allows to summarize two kinds of algorithms, strategies, methods, tools and general concepts. The first kind have the shared objective to support the creation of technical systems that are fault-tolerant as a consequence of their diagnosability and controllability. The second kind have the shared objective to support the creation of technical systems that are fault-tolerant as a consequence of the inherent fault-tolerant design qualities of these technical systems [4]. Up to today, only a small number of research initiatives in this field can be identified, and the orientation in this field is anything but easy. The outcomes of research activities in the field of integrated product development and systematic design [35,36,37,38,39] can serve as a basis for further research activities. Until now, FTD was not a main focus in this research field; only a small amount of research works was directly concerned with FTD. The enormous importance of this research field is expressed by Rouissi and Hoblos [40]; they underline that the ability of a system to accommodate faults has to be achieved employing a conscious FTD. A considerable number of FTD approaches especially in the field of electronics engineering are complied in [41]. Initial research works that concern smaller investigation fields focus on wireless sensor networks [42], on voting logic and redundant actuation devices [43], on chip design [44], on design of frequency converters [45] and on artificial intelligence [46]. An approach for structuring this field is proposed by Stetter [4]. In this publication, a structure for FTD is proposed, which is based on the established models of product concretization in design and product development science—for instance, by [38].

**Figure 2:**
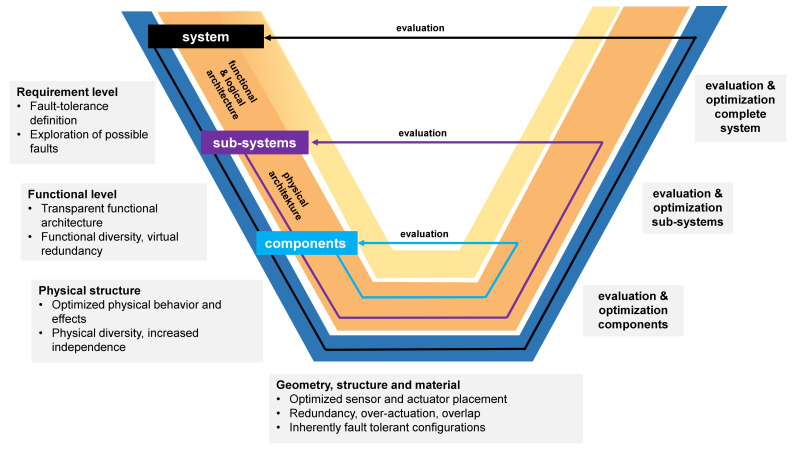
Levels for characteristics of fault-tolerant design according to the levels of product concretization depicted in a V-model.

Today, most technical products include elaborate software functions and need to communicate with other technical products or super-ordinate systems, e.g., via cloud services, i.e., those are cyber-physical systems (CPS). For the development of such systems the VDI/VDE-guideline 2206 [47] is proposed, which was recently developed from an earlier guideline describing the product development of mechatronic systems. A central concept of this guideline is the well-know V-model that describes the transition from requirements to a final product. This model is intended to guide product development engineers in their processes and allows to assign product concretization levels, which are prevailing in the different logical sub-areas of the whole product development process. The different levels for the characteristics of FTD are represented in Figure 2—adapted from [4].

On all levels, the search for possible faults is one of the essential elements of FTD:

- Requirement level: Usually already in the earliest stage certain components, e.g., certain sensors, are already predefined, e.g., because of legal obligations. Usually, possible faults of these components are already known. Additionally, the collection of requirements is accompanied with some kind of benchmark with the predecessor product or competing products. Usually, the benchmark analyses will also produce possible faults.
- Functional level: Frequently, faults are caused by an unfavourable interplay of components. This interplay can be investigated on a functional level and can by employed to search for possible faults.
- Physical structure: Certain faults are connected not only with certain components, but with certain physical phenomena. All kinds of optical sensors, for instance, are susceptible to contamination. Consequently, an analysis of the physical phenomena can also by used for searching for possible faults.
- Geometry, structure and material: On this level, the search for possible faults will concentrate on the applied sensor and actors; even a quantitative evaluation is often possible, because certain values such as the mean time between failure (MTBF) or the reliability in terms of failure rate λ are known. Detailed investigations are possible employing methods such as failure mode and effects analysis (FMEA), fault tree analysis (FTA) or event tree analysis (ETA).

The most important concept of FTD is the employment of redundancy, i.e., the multiplication of certain entities in order to increase reliability. The most common example are multiple engines on an airplane, which allow at least a safe landing, even in the case of the failure of one engine. It is important to note that redundancy can not only mean the duplication, triplication, etc., of components of a technical system, but also the addition on other levels:

- Functional level: A technical system can be equipped with redundant entities with functional diversity, i.e., physical and non-physical subsystems, which fulfil the same function. One example can be the combination of a “real” physical sensor and a virtual sensor, which creates a sensor signal by means of a mathematical model, i.e., an analytical redundancy.
- Physical structure: A technical system can be equipped with redundant entities with physical diversity. A typical example is sensors which are based on different physical phenomena, e.g., a combination of a optical sensor with an ultrasonic sensor and the application of a sensor fusion algorithm,
- Geometry, structure and material: On this level, a direct multiplication of components, frequently sensors and actors, is possible.

In the next section, the main design characteristics concerning FTD for the AGV are discussed in a sensible sequence according to the model shown in Figure 2, and the different elements of Figure 2 are also explained in this context.

## 3. Characteristics of Fault-Tolerant Design

### 3.1. Fault-Tolerant Design on the Requirements Level

A general definition of requirements describes them as the goals, purpose, constraints and criteria that are directly connected with the product development process of a technical system [48]. Today, the fact is widely accepted that requirements are one of the most important factors in industrial product development and that a sensible management is crucial; compare for instance [49]. In industrial systems development, four of the ten top risks in projects are directly associated with requirements [50]. Detailed investigations of industrial product development projects were able to show that only 52 percent of the originally defined requirements will really appear in the final produced version of the system [51].

One main prerequisite for the product development of complex technical system is an explicit and conscious Requirements Management (RM) [52,53]. Central elements of RM are the identification, investigation and collection of requirements, their definition and documentation, the building of consensus of the involved stakeholders, the validation of the requirement fulfillment and the definition of measures for controlling and managing these requirements [52]. For higher complexity systems, tools which support RM are necessary; one example frequently applied in industry is the IBM Engineering Requirements Management DOORS Family [54]; (DOORS is a product of IBM cooporation, Armonk, NY, USA). Meanwhile, a comparable amount of functionality is also available in open source tools such as Eclipse ProR [4].

Ongoing research activities are focused on the integration of RM in a digital product life-cycle; a substantial integration may be for instance realised by means of applying graph-based design languages that are based on UML [55]. A main prerequisite for FTC as well as FTD is a RM including monitoring requirements. The investigation of requirements is an crucial step of FTD. This investigation has to include all thinkable fault possibilities, including expected faults and probable faults and it has to include the level of fault-tolerance that needs to be achieved as well as the form and amount of redundancy that needs to be realised [4].

### 3.2. Design Characteristics on the Functional Level

**Figure 3:**
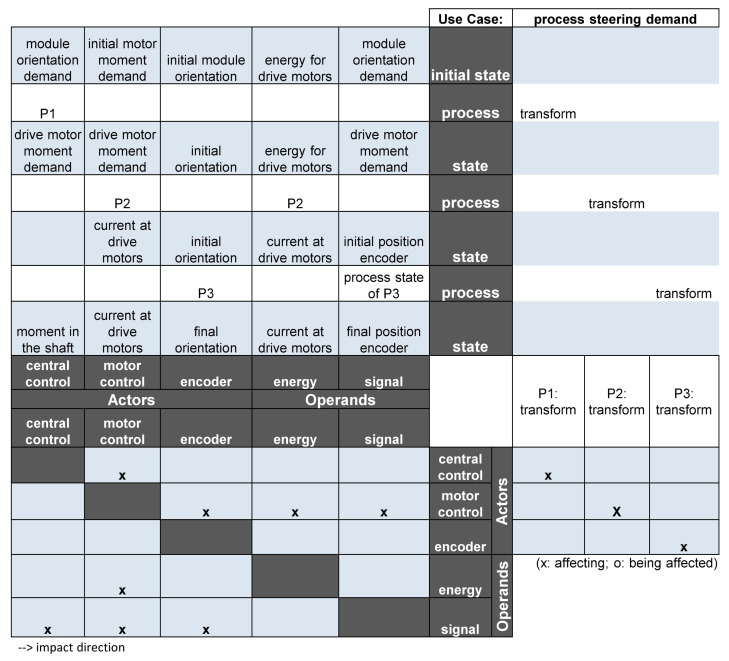
Functions of an AGV modelled in the IFM framework.

One cornerstone of a successful product development of a technical system is a detailed knowledge concerning the different functions of the system [56]. Functions can be connected with the direct purpose of the system, e.g., for a AGV, the central function is to transport certain goods. However, much more functions are realised in such a system such as a steering function, control functions and diagnosis functions. Such functions are described on the so-called “function level”, which is the most abstract level of product description that describes the technical solutions appropriate to enable the fulfillment of functional requirements. A considerable number of research activities are concerned with this rather abstract level [56]. In this scope, a synoptic framework was developed in recent years—the Integrated Function Modelling framework (IFM)—and approaches were investigated to integrate it in digital development processes of technical systems [57]. Figure 3 shows a central functionality of an AGV modelled using IFM.

In this figure, a certain use-case of the AGV is modelled—the processing of a steering demand. This function will be revisited in the section concerning characteristics on the physical level. The IFM contains a state view (upper left), a process flow view (upper right), an interaction view (lower left) and an actor view (lower right). The integrated combination of these views allows a comprehensive modelling of the functional relationships in a technical system. This combination and integration in an engineering framework also support the development of fault-tolerant design characteristics [58].

**Figure 4:**
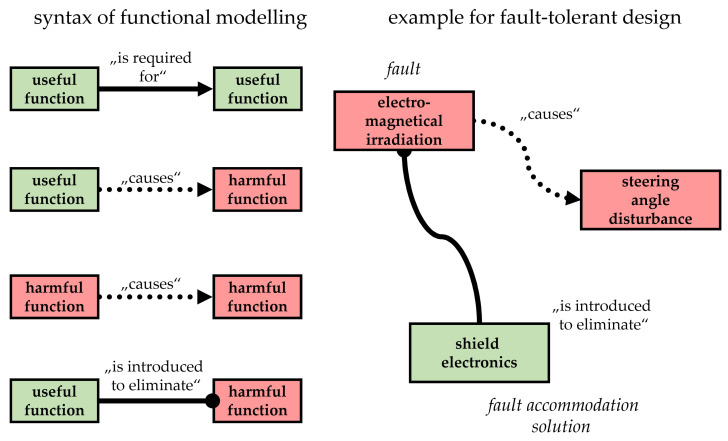
Accommodation of the fault “steering angle disturbance”.

On the product concretization level of functions, the analysis of faults and their consequences deserves special attention. Additionally, functional possibilities to accommodate them can be investigated. In this endeavour, control and diagnosis functionalities are worthy of special attention. In this case, the application of a relation-oriented function modelling technique may be helpful. This kind of function modelling technique allows to distinguish between useful and harmful functions and is based on modelling methods proposed by the research community which employs the theory of inventive problem solving (TIPS/TRIZ) (compare, e.g., [59]). Figure 4 shows the syntax of function modelling when employing this together with a simplified example concerning the considered AGV.

The fault described in Figure 4 concerns a problem in the product development and testing of the AGV. This problem was characterised by disturbances in the sensor signal of the encoder, which measures the angular position of the driving module. It is obvious in this function model that the respective fault “electro-magnetical irradiation” is a harmful function that is causing another harmful function “steering angle disturbance”. In this kind of function model, one may document the fact that the functional solution “shield electronics” is introduced as a useful function to eliminate the consequences of this fault and therefore to realize fault accommodation.

**Figure 5:**
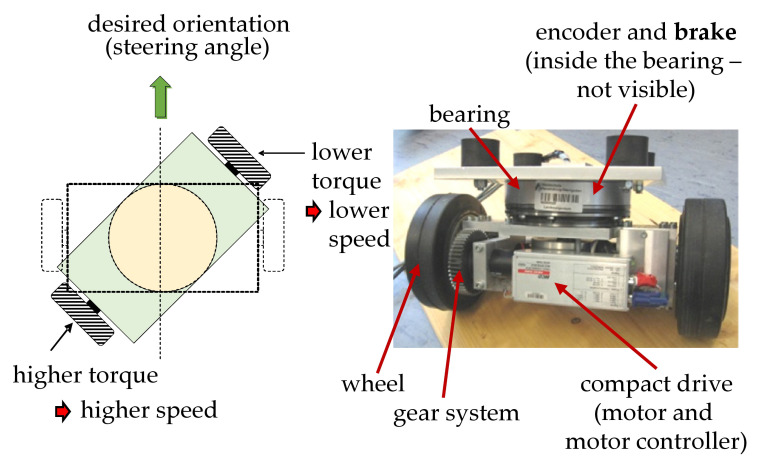
Drive module of the AGV—torque steering and certain components.

FTD on the function level can be achieved by several means; one example with high potential are analytical redundancies (compare [4]). Analytical redundancies are more than one functional entity which fully a certain function. Commonly, this will be a monitoring function. Due to the separation from any sensory measuring needs, redundancy on the function level is the highest and most independent form of redundancy. Based on the given example of the AGV, a fuzzy actuator, which is based on an analytical redundancy—a virtual sensor, is elucidated in this section [60]. As mentioned above, the considered AGV disposes of four mechanically independent drive modules. The basic concept of torque steering and certain components these drive modules is shown in Figure 5.

The desired orientation, i.e., a certain steering angle, can be achieved through balancing the torque of two drive motors that are both connected by means of a gear system to one wheel. In a theoretical consideration, one would assume that two drive motors, which are assigned the same task would cause redundancy and thus an increased fault-tolerance. Nonetheless, in the given application the AGV could not drive, if one of the drive motors fails, because the balancing of torque would not be possible. An analysis of the higher levels of product concretization (compare Figure 2) can pave the way to another possibility: inside the main bearing, which connects the drive module to the AGV frame, a brake was installed (Figure 5). This brake is able to prevent the rotation possibility of the drive module, i.e., to lock the steering module. During standstill of the AGV, the torque generated by only one drive motor can be employed to accomplish the desired orientation and, after locking the module with the brake mentioned above, driving in the predetermined direction is possible. Consequently, up to four faults of drive motor in the four different drive modules can be accommodated. This capability considerably enhances the fault-tolerance of the AGV. Additionally it was found that these brakes improve the dynamic behaviour of the AGV when driving with high velocity in a constant direction. Consequently, the addition of the steering brakes may also be understood as an instance of over-actuation (compare [4]).

On the functional level, FTC may be able to guarantee a safe operation of the AGV, even when a fault occurs. In the given example, it is possible to realize FTC by employing a fuzzy virtual actuator. In this kind of virtual actuator, the application of fuzzy logic rules leads to the integration of the knowledge of experts into decision making [60]. Using an in-depth dynamics investigations and discretizations techniques, the vehicle can be described in subsequent form:

(1) $$G_{k}x_{k+1}=G_{k}x_{k}+B_{k}u_{k}+E_{k}d_{k}+Ww_{k},$$

In this equation, $w_{k}$ denotes an exogenous disturbance vector and W denotes the respective distribution matrix. $G_{k}$, $B_{k}$ and $E_{k}$ stand for the system matrices.

These matrices are derived from the design of the AGV, from kinematic and kinetic evaluations and from expert’s knowledge. The considerations started with a detailed description of the kinematic parameters and an analysis of the forces acting on all eight wheels of the AGV. From this the force leading to a longitudinal motion as well the lateral could be derived. A consideration of the dynamic behaviour (Newton’s second axiom) allowed us to calculate the required torque of each of the drive motors, and the yam rate dynamics could be evaluated. This allowed the elaboration of a state space model [60].

**Figure 6:**
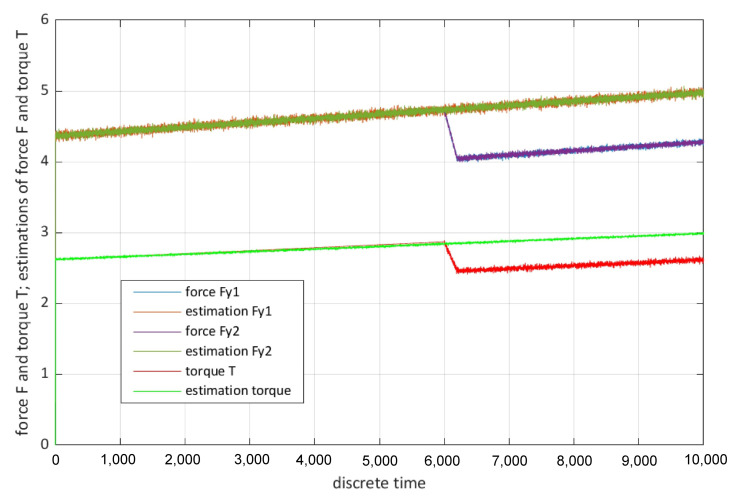
Exemplary estimation result; fault at k = 6000 s.

The first stage in the application of this virtual actuator is the detection of possible faults, in this stage a virtual sensor that employs an unknown input estimator (UIE) may be used [60]; Figure 6 shows an exemplary estimation result of this input estimator.

**Figure 7:**
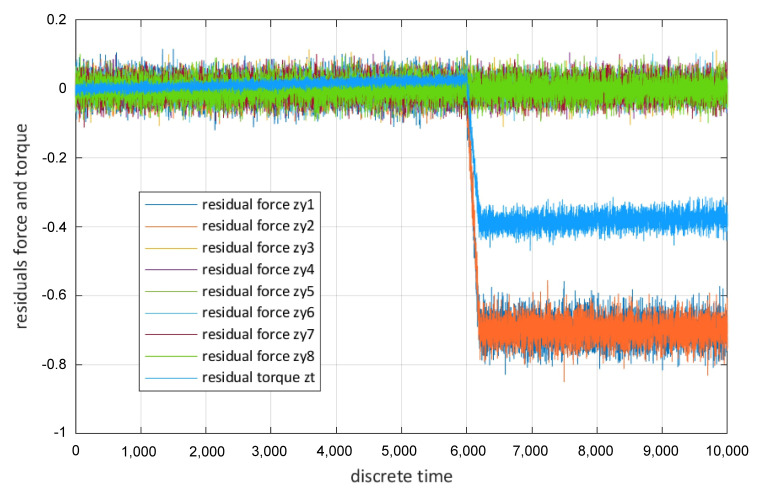
Residuals generated by the virtual sensor.

The employed UIE is based on a recursive filter similar to the filter developed by Gillijns and De Moor [61]. Visible in Figure 6 are longitudinal forces, which are caused by the driven wheels and their estimations generated by the UIE as well as the total torque acting on all wheels and its estimation. At k = 6000 s the consequences of a fault are visible—a slippery surface under one driving module. By comparing the estimations with sensor signals, residuals can be generated, these residuals are shown in Figure 7.

These residuals are the input information for a fuzzy virtual actuator. The output of a fuzzy virtual actuator is a compensation factor that can be applied to sensor readings for allowing the original controller to control the AGV drive motors in case of a fault [60]. It is important to note that the residuals can also be combined with elements of the state as input for the fuzzy virtual actuator [62]. For each residual $z_{m,j}$ three membership functions $\mu_{z_{m,j}}$ are derived. These three membership functions allow the initial evaluation of the individual residual. In prior research, it was established that trapezoidal membership functions are appropriate in the area of residual evaluation [63]. An example for a membership function is the first input membership function $\mu_{m,j,1}$ [60]:

(2) $$\begin{array}{c} \mu_{m,j,1}=\left\{\begin{array}{cc} 1, & z_{m,j}<c_{m,j,1}\\ \frac{d_{m,j,1}-z_{m,j}}{d_{i1}-c_{m,j,1}}, & c_{m,j,1}\leq z_{i,j}\leq d_{m,j,1}\\ 0, & z_{m,j}>d_{m,j,1} \end{array}\right. \end{array}$$

**Figure 8:**
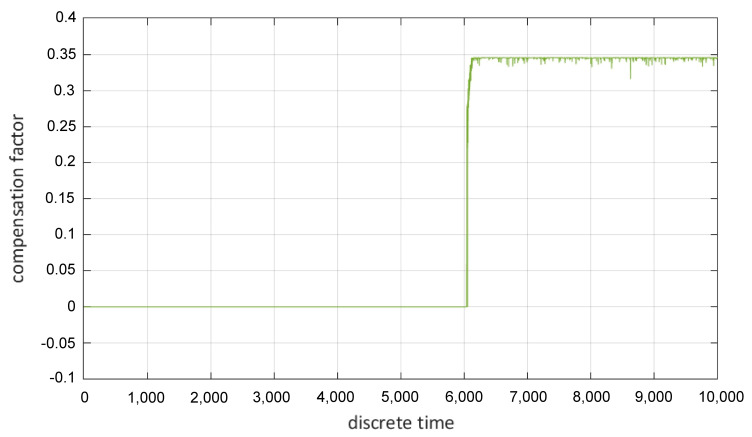
Compensation factor generated with the fuzzy virtual actuator.

In these equations, $c_{m,j,1}$ and $d_{m,j,1}$ stand for parameters that are determined based on experimental data and/or expert’s knowledge. The FIS also disposes of appropriate output functions (compare [60]), which lead to a sensible compensation factor. This compensation factor is the output of the fuzzy virtual actuator (Figure 8).

It is also obvious that the presence of the fault immediately leads to a changed and stable compensation factor; thus underlining the effectiveness of the proposed fuzzy virtual actuator. The implementation of the specific realization of an analytical redundancy consequently presents an appropriate function level measure intended to increase the fault-tolerance of the AGV. The novelty of this approach in comparison with known fault-tolerant control theory is the application of a fuzzy inference system (FIS), which combines the analysis of residuals and state variables and allows us to incorporate the knowledge of experts.

### 3.3. Design Characteristics on the Physical Level

**Figure 9:**
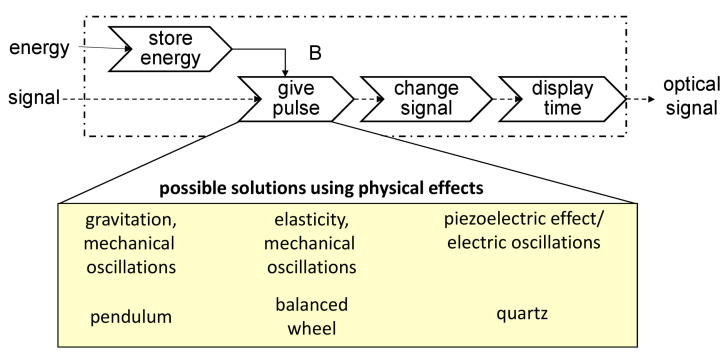
Example for physical phenomena for the realization of a function of a watch.

Current research activities underline that representations of abstract physics are more and more important for the product development of technical systems [55,64]. This level of abstraction is a bit more concrete than the function level. On this level, it is described how a desired functionality of a technical system can be realized by employing certain physical (and chemical) phenomena. For the sake of further clarifying the notion of “physical phenomena”, examples are represented in Figure 9 using the example of watch [64].

In the upper section of Figure 9, a functional view of a watch is visible. This view contains the main functions “store energy”, “give pulse”, “change signal” and “display time”. These functions transform energy and an initial signal into an optical signal. One of these functions, ”give pulse” can be realised using different physical phenomena such as gravitation and mechanical oscillations by means of a pendulum in an old-fashioned wall-mounted clock. Other possibilities are elasticity and mechanical oscillations as in a mechanical watch or the piezoelectric effect and electric oscillations in a common quartz watch.

**Figure 10:**
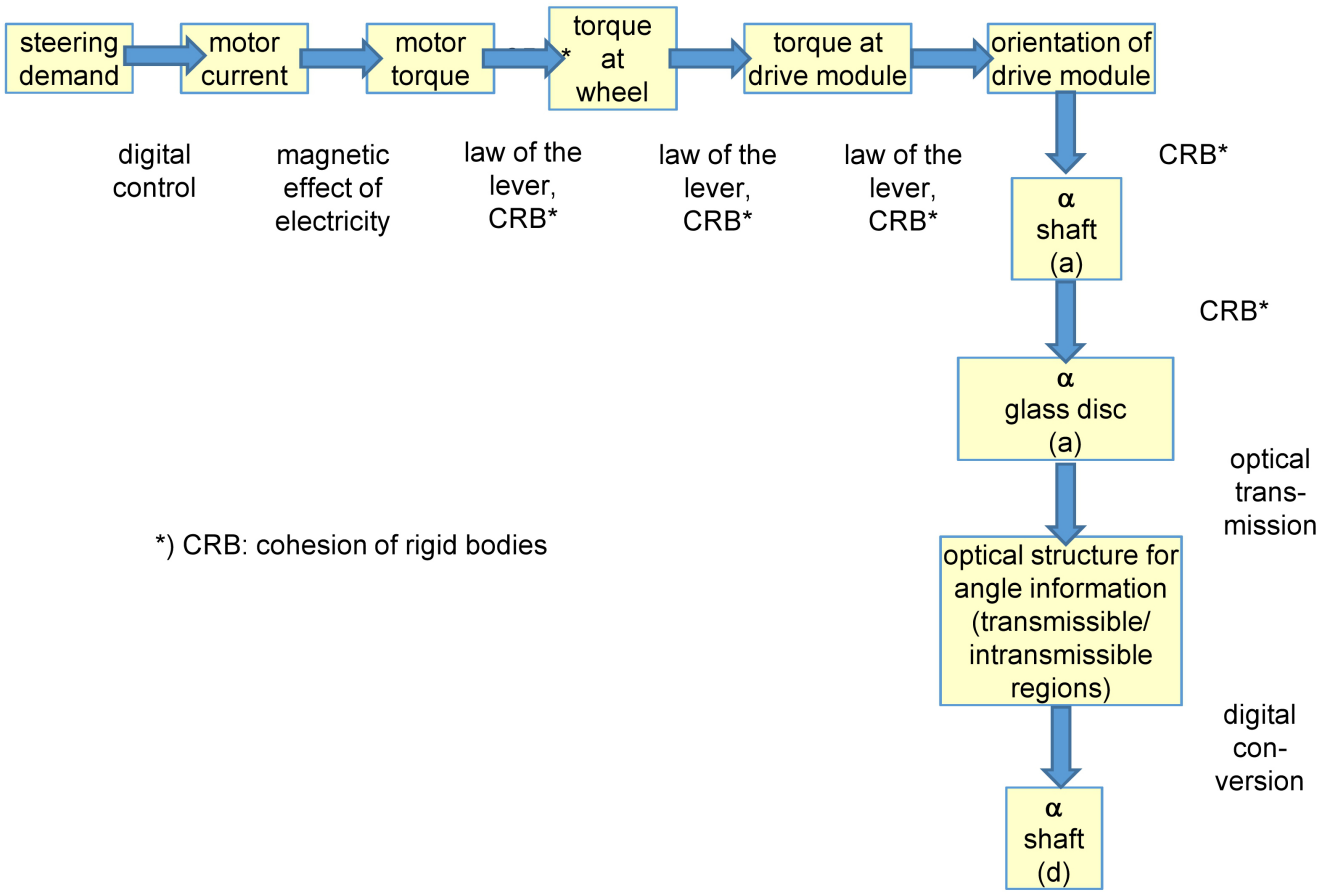
Physical effect chain for the steering system of the AGV.

The exploration of the physical phenomena is especially interesting concerning the steering system of the AGV. In this kind of AGV, no real, physical steering system is present, but the steering demands are realized by means of different speeds of wheels on drive modules and the angular orientation of the drive modules which is a consequence of the torque steering principle explained earlier. How is this kind of steering demand processed in this kind of AGV? The elementary physical phenomena or “effects” that realize this functionality are represented in Figure 10.

This effect chain starts with an expressed steering demand. A pseudo effect “digital control” enables the transformation into motor “currents” at the different drive motors. In these motors, the effect “magnetic effect of elctricity” leads to the generation of a torque at the motor output shafts. Two physical effects—“law of the lever” and “cohesion of rigid bodies”—in the gear system attached to the motor lead to the generation of torque at the wheels. The same physical effects allow the orientation of the drive module. The physical effect “cohesion of rigid bodies” allows to transform this wish into a angle information concerning the vertical shaft in the drive modules. This angle is then measured by means of an encoder using the physical effects ”optical transmission” and ”digital conversion” (shown in the lower right of Figure 10).

In the considered AGV, redundant entities with physical diversity can be found in the on-board localization system. The special design of the drive system allows, in contrast to AGV or robots with mecanum wheels, a good orientation employing odometry. In this context, odometry means to measure the angles that the different wheels have turned and to measure the steering angles and to apply a kinematic model of the robot in order to follow position changes of the AGV. This position determination relies on the process of dead reckoning, which is the ongoing approximate determination of the location of a moving object based on the direction of movement and velocity. Still, a physically different entity is present in the form of ultrasonic sensors at the side of the AGV. These sensors would avoid collisions, even if a fault leads to a wrong odometry result. Additionally, these sensors are used for aligning the AGV and for the orientation on landmarks.

In accordance with literature [36], it can be found that the representation on an abstract physical level allows a profound discussion without narrowing the space of possible solutions too much. The behavioural abstract physics model fosters a deeper understanding and consequently supports FTD. Based on this understanding, solution elements for increasing the fault-tolerance could also be found on the most concrete level—the level of geometry, structure and material.

### 3.4. Design Characteristics on the Level of Geometry, Structure and Material

**Figure 11:**
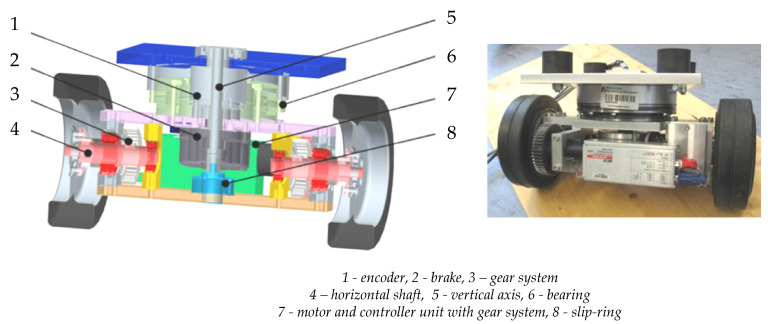
Components of the drive module of the AGV.

The physical appearance of technical systems includes the geometry of all components, the material(s) they are made of, the characteristics of the surfaces and the structure of these components, i.e., their spatial arrangement in sub-systems and modules. The complete appearance is described on the most concrete level of the model shown in Figure 2. On this level, designers need to describe everything that is necessary to produce and operate the technical system in an unambiguous manner. All measures concerning an increase of fault-tolerance, which rely on a duplication of elements, need to be described on this level [4]. In this paper, the discussion will again concentrate on the AGV. It was highly desirable to come up with a fault-tolerant design of the AGV, because an inoperable AGV in a warehouse would considerably decrease the efficiency of the whole system. A first instance of redundancy can be found in the slip-ring in each module which is responsible for transferring energy and information from the AGV frame into the module which can turn freely; the components of a drive module of the AGV are illustrated in Figure 11.

This slip-ring is visible as position 8 in Figure 11. A cable through the hole in the vertical axle 5 is connected to the upper part of the slip-ring. The lower part rotates with the module and the cable from this part supplies all components within the module with energy and information. In the given application a slip-ring which can transfer 15 distinct lines was chosen even in the case that only a controller area network (CAN) bus and a single supply voltage of 24 V was needed (4 lines—the information from the encoder was also transmitted via the CAN bus). All signals were transferred over more than one ring in order to increase both reliability and transferable current.

**Figure 12:**
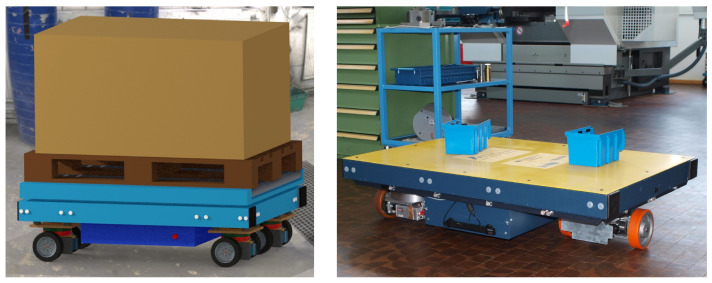
Redundant ultrasonic sensors at the side of the AGV.

Another instance of redundancy are the ultrasonic sensors on all sides of the AGV (Figure 12).

Each long side was equipped with four sensor pairs in order to achieve a certain amount of sensor overlap (compare [4]) and to increase the reliability. The redundancy of the relatively light and cheap elements consequently increased the fault-tolerance. Visible on the right side of Figure 12 is also an earlier version with only three drive modules, which can also perform all manoeuvrers. In an AGV of this kind with four drive modules also some kind of redundancy is present.

A measure—or design characteristic—which is also frequently recommended for increasing the fault-tolerance of technical systems is over-actuation. Usually, the term “over-actuation” describes the integration in the design of technical systems of more actuators than actually necessary for controlling the involved motion systems [65,66]. Additionally, the term over-actuation can also denote the use of stronger actuators than necessary for realizing certain motions [4]. This kind of system design leads to over-actuated technical systems, which dispose of a superior controllability. Additionally, this kind of system design may increase the fault-tolerance, because the over-actuation potential can be used for the compensation of the effects of faults [4]. Coming back to the design of the AGV it is important to note that a certain amount of over-actuation—realized in the drive motors—is inevitable for achieving a satisfactory controllability. This amount of over-actuation is also inevitable for allowing the compensation of possible faults, e.g., a slippery surface. For the general design of such systems, which may only function if a nearly perfectly working control system is present, a large amount of fault-tolerance is mandatory, also because some potential user mistakes cannot be predicted [41].

## 4. Conclusions and Outlook

In the product development of complex technical system, designers need to strive for a large amount of fault-tolerance in order to reduce the sensibility of the system to faults, which may be caused by unexpected operation and environment conditions, by manufacturing and assembly imperfections of sensors and actuators as well as by unexpected user activities. This paper concentrated on different facets of a conscious fault-tolerant design. Different approaches and measures were presented based on the example of an AGV, and it was explained how they can contribute to an increased fault-tolerance. The discussion was based on a model using the different levels of abstraction of the product description for giving a structure for ordering the different measures of fault-tolerant design. This model allowed the discussion of several design principles and concrete approaches. The example also served to clarify that a holistic approach for increasing the fault-tolerance of technical systems is needed and that this approach goes beyond a rather simplistic addition of redundancy on a concrete level. The experience in the design and realization of the AGV made clear that it is of paramount importance to connect the algorithm development with the system development. A key element for a high level of fault-tolerance is a good controllability. A good controllability is usually the result of a strong interconnection between sensible design characteristics and powerful control algorithms and is highly desirable. Another important factor is fault detection and identification. Again, a strong interconnection between conscious design characteristics and diagnosis algorithms was found to be advantageous. It is possible to conclude that a combination of fault-tolerant control with consciously developed design characteristics also intended to increase the fault-tolerance may lead to technical systems with a superior behaviour in the case of faults.

One important part of the scientific contribution is the methodological framework for fault-tolerant design. The main advantages of this framework are a differentiated modus operandi on the different level of product concretization. The opportunities, restrictions and uncertainties of certain measures to accommodate faults can be analysed in detail and the search for dedicated solution elements can be supported. A challenge is the integration in the product development process; engineers already need to consider a large variety of different aspects, and fault-tolerant design is an additional one. However, early and conscious considerations of the aspects of FTD are more likely to lead a robust and economical product.

Another important part of the scientific contribution are the individual algorithms and methods for fault-tolerant design. On an intermediate level—the functional level—a virtual actuator is proposed and described in this paper, which allows the generation of correction factors for the control system. On another intermediate level—the physical level—the advantages of a transparent physical structure were demonstrated.

Obviously, the reported observations and conclusions are, so far, based on a limited number of case studies. Further research is needed and already planned, which will focus on an expansion of the knowledge basis of fault-tolerant design.

## Abbreviations

The following abbreviations are used in this manuscript:

AGV	Automated guided vehicle
CAN	controller area network
CPS	Cyber-physical system
DC	Direct current
ETA	Event tree analysis
FMEA	Failure mode and effect analysis
FTA	Fault tree analysis
FIS	Fuzzy inference system
FTC	Fault-tolerant control
FTD	Fault-tolerant design
IFM	Integrated function modelling
MBSE	Model Based Systems Engineering
RM	Requirements Management
TIPS	Theory of inventive problem solving
UIE	Unknown Input Estimator
UML	Unified modelling language
